# Effect of malaria chemoprevention for school-age children across transmission archetypes: a modelling study

**DOI:** 10.1016/S2214-109X(25)00325-0

**Published:** 2025-11-12

**Authors:** Joshua Suresh, Marita Zimmermann, Catherine Maiteki, Anne Stahlfeld, Abigail Pratt, Don P Mathanga, Sarah G Staedke, Miriam K Laufer, Chris Drakeley, Caitlin Bever, Lauren M Cohee

**Affiliations:** a Institute for Disease Modeling, Gates Foundation, Seattle, WA, USA; b Ministry of Health, Kampala, Uganda; c The Feinberg School of Medicine, Northwestern University, Chicago, IL, USA; d Gates Foundation, Seattle, WA, USA; e Malaria Alert Centre, Kamuzu University of Health Sciences, Blantyre, Malawi; f Liverpool School of Tropical Medicine, Liverpool, UK; g Center for Vaccine Development and Global Health, University of Maryland School of Medicine, Baltimore, MD, USA; h London School of Hygiene and Tropical Medicine, London, UK

## Abstract

**Background:**

Intermittent preventive treatment (IPT) of school-aged children with antimalarial drugs decreases rates of infection, anaemia, and clinical malaria. Since school-aged children are a major transmission reservoir, we estimated the effect of IPT for this group on *Plasmodium falciparum* transmission to younger children and adults across three epidemiological settings.

**Methods:**

With the use of an established malaria transmission model, we developed three epidemiological archetypes (Sahelian, Central, and Southern African) and estimated the effect of IPT of school-age children across transmission levels (*P falciparum* parasite rate in children aged 2–10 years [*Pf***PR**_2–10_]: 5–40%). Long-lasting insecticide-treated nets and clinical case management were always included as baseline interventions. We compared scenarios for three of the most widely studied drug options (dihydroartemisinin–piperaquine, artesunate–amodiaquine, and sulfadoxine–pyrimethamine–amodiaquine) and delivery options (school-based or community-based), estimating clinical cases averted.

**Findings:**

When long-acting drugs were administered frequently (monthly campaigns with dihydroartemisinin–piperaquine), modelled IPT of school-age children averted 70–90% of cases in school-aged children (up to about 2·0 cases per child per year) and 20–60% of cases in younger children and adults (up to about 0·5 cases per person per year), with greater community benefit at lower transmission levels (*Pf***PR**_2–10_ 5–10%). Shorter-acting drugs (sulfadoxine–pyrimethamine–amodiaquine in the Sahelian archetype or artesunate–amodiaquine in the Central and Southern archetypes) administered monthly or longer-acting drugs administered once per school term averted 40–60% of cases in school-aged children (up to about 1·3 cases per child per year) and 15–50% of cases in other ages (up to about 0·5 cases per person per year).

**Interpretation:**

Our model suggests that adding IPT of school-age children to current control tools could decrease malaria burden in this group and reduce *P falciparum* transmission.

**Funding:**

US National Institutes of Health, Doris Duke Clinical Scientist Development Award.

## Introduction

Global progress towards malaria eradication is stalling, especially in sub-Saharan Africa where annual clinical incidence has plateaued. Further progress will require improved targeting of subpopulations at high risk of malaria.^1^ Across sub-Saharan Africa, and particularly in areas where malaria control is faltering, children aged 6–15 years (school-aged children) are the largest reservoir,^2^, ^3^, ^4^ with age-stratified *Plasmodium falciparum* prevalence peaking in this age group.^5^, ^6^, ^7^, ^8^, ^9^, ^10^ School-aged children are not only a link in onward transmission but also experience negative effects from malaria. Although historically less than 10% of malaria-related mortality occurs in children in this age group,^11^ frequent malaria infections, both acute and chronic, contribute to morbidity and anaemia, which decrease cognitive function and educational attainment.^12^, ^13^, ^14^ Despite the need for interventions to protect school-aged children, this group benefits least from current control interventions. Compared with younger children and adults, school-aged children are less likely to sleep under bednets;^15^ they are also less likely to seek treatment and receive effective case management since many infections in this age group do not result in severe symptoms.^16^ Moreover, vaccination and chemoprevention programmes, including seasonal malaria chemoprevention and perennial malaria chemoprevention, primarily target younger children.

Intermittent preventive treatment (IPT) in school-aged children might address this key gap in malaria control. In June, 2022, WHO issued a conditional recommendation endorsing IPT in school-aged children as an effective approach to reduce infection, clinical malaria, and anaemia in this group.^17^ This recommendation is supported by findings showing the clinical effect of IPT on individual children.^18^ Although the evidence base is strong, it is limited by heterogeneity of intervention design, study setting, and results among the studies. Key outstanding research questions include the following. Which antimalarial drugs should be administered with what frequency? What level of community benefit can be expected based on reduction in transmission from school-age children? And how does effect vary in different transmission settings and levels?

**Research in context**

**Evidence before this study**

We searched PubMed for publications, without language restrictions, from Jan 1, 2000, until March 1, 2024, with the search terms “malaria” AND (“school”[text word]) AND “modeling” AND “treatment”. No previous modelling studies or clinical trials have evaluated the effect of intermittent preventive treatment (IPT) of malaria in school-age children on transmission across different transmission settings. One previous cluster randomised clinical trial by Staedke and colleagues evaluated the effect of IPT of school-age children with dihydroartemisinin–piperaquine administered monthly in schools in Uganda. The trial found a small but statistically significant reduction in community parasite prevalence. However, the magnitude of the effect was likely limited by low intervention coverage. In Senegal, Cisse and colleagues found a reduction in clinical malaria in older children and adults after implementing seasonal malaria chemoprevention in children younger than 10 years. Stuckey and colleagues modelled the effect of test-and-treat campaigns administered to school-aged children once or twice per school term in a low-transmission site in western Kenya, with the use of artemether–lumefantrine for treatment. More recently, Runge and colleagues modelled IPT of school-aged children in medium-transmission and high-transmission zones of Tanzania, with the use of a hypothetical drug with a 14-day prophylactic duration, administered twice per year. However, since these studies each focused on a single IPT of school-aged children protocol and a single transmission setting, it is challenging to generalise their findings to other settings and other potential designs of IPT in school-aged children.

**Added value of this study**

In this study, we modelled multiple approaches to IPT of school-aged children considering different antimalarial drugs, frequencies of administration, and modes of delivery (ie, school-based or community-based). We evaluated the effects on both school-aged children themselves as well as younger children and adults in three African transmission archetypes and across a range of transmission levels within each archetype. Our models address key questions identified as knowledge gaps in the WHO conditional recommendation for IPT of school-aged children, including alternative drug regimens based on level of transmission and effect on community-level transmission.

**Implications of all the available evidence**

School-aged children bear a considerable burden of malaria and are the predominant reservoir of *Plasmodium falciparum* transmission in sub-Saharan Africa. Our models show that well designed IPT interventions for school-aged children can reduce clinical malaria in this group and decrease transmission to younger children and adults across diverse settings. These findings suggest that IPT of school-age children is a promising complement to existing malaria control strategies.

Mathematical models of malaria transmission offer a low-cost way to explore intervention implementation and effect, which can then be further investigated through rigorous large-scale clinical trials. Recently, mathematical models have been used to estimate the benefit of improved rapid diagnostic tests,^19^ to project the population-level effect and cost-effectiveness of vaccines,^20^ and to evaluate the inclusion of systemic endectocides in mass drug administration campaigns.^21^ Previous modelling studies of specific approaches to target school-aged children in single transmission settings predicted a modest reduction in malaria cases across all age groups.^22^, ^23^ No previous modelling studies have compared intervention designs of IPT in school-aged children across transmission settings.

To address the pressing epidemiological and operational questions around IPT of school-aged children, we used a comprehensive model of malaria transmission to explore potential effects of this treatment under different intervention protocols and transmission archetypes. We examined drug choice, delivery approach, and campaign frequency of IPT in school-aged children; estimated both the direct effect of the intervention on school-aged children and the indirect effect of reducing transmission; and compared optimal school-based chemoprevention with further scale-up of currently available malaria control interventions. Results are intended to support the design of rigorous clinical trials, develop further implementation guidance, and inform prioritisation of IPT in school-aged children among malaria control interventions.

## Methods

### Model structure and set-up

All simulations were done with the epidemiological modelling software (EMOD; version 2.20).^24^, ^25^ In EMOD, humans are modelled as individual agents and vector populations are modelled as discrete cohorts based on lifecycle stage, infection status, and infectiousness. A within-host model simulates the dynamics of human malaria infections, predicting parasite densities and likelihood of symptom presentation based on past exposure and acquisition of immunity. Previous work has extensively calibrated EMOD model parameters to broadly reproduce age-stratified prevalence, incidence, severe disease incidence, asexual and gametocyte densities, and infectiousness across numerous sites spanning a wide range of transmission intensity and seasonality.^26^, ^27^, ^28^ To model IPT of school-aged children under realistic transmission conditions, EMOD was run within different transmission archetypes and assumed pre-existing baseline interventions. Key baseline assumptions and input parameters are summarised in table 1.

**Table 1:** Summary of key input parameters in analysis

	**Assumed value**
Health-seeking rate	60% for children younger than 5 years and 30% for older individuals
School attendance	80% of children aged 6–12 years^29^
Baseline ITN coverage	70%
Treatment regimen adherence for school-based IPTsc	95%
Treatment regimen adherence for SMC	80%
Fraction of school-attending children who receive IPTsc during a round of school-based IPTsc	90%
SMC per-round coverage	60%
Drug efficacy	Drug-specific pharmacokinetic and pharmacodynamic parameters were determined through calibration to data; drug efficacy is a byproduct of these parameters (appendix p 5)

INT=insecticide treated net. IPTsc=intermittent preventive treatment of school-aged children. SMC=seasonal malaria chemoprevention.

Initial population immunity distributions were generated by running a 50 year burn-in simulation starting with an immunologically naive population of 5000 individuals. Birth and death rates were balanced to maintain a roughly constant population size whose age structure approximates that of sub-Saharan Africa. During the burn-in period, bednet coverage and case management rates gradually increased. The burn-in endpoint marks the beginning of the scenarios of interest. Every model scenario was simulated for 2 years, and the impact assessment is done in the second year. This time window was chosen to capture the effect on transmission after IPT of school-aged children has been fully rolled out, rather than the short-term changes that occur during initial roll-out of the intervention (see appendix (p 13) for an examination of the effect on a much longer time window).

This modelling study did not require ethical approval.

### Setting and location

**Figure 1:**
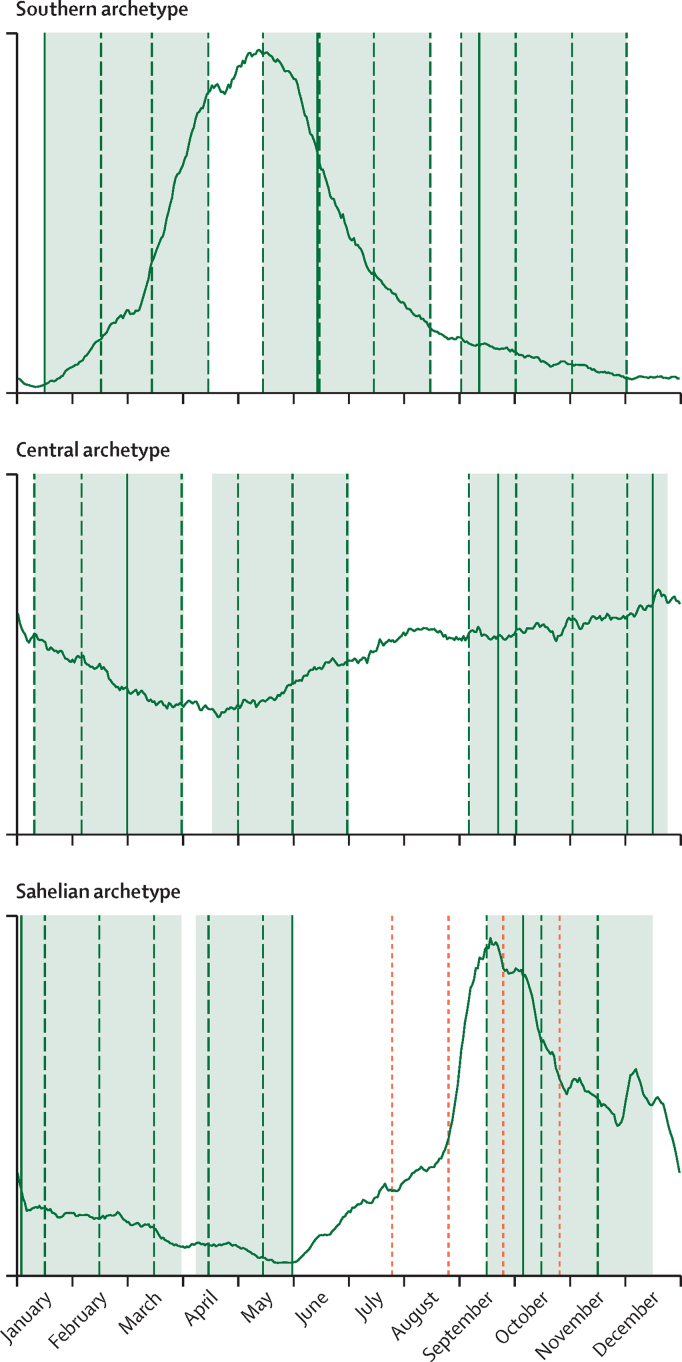
Seasonality and school calendar for each of the three modelled transmission archetypes X-axis is month of the year. The y-axis represents the relative seasonality of clinical cases (unitless). Solid black curves show the seasonality of new clinical cases in the model, when simulated with *Plasmodium falciparum* parasite rate in children aged 2–10 years of approximately 10%. Shaded green areas show the assumed academic calendar when primary school is in session; school-based campaigns of intermittent preventive treatment (IPT) of school-aged children are restricted to occur only during these time windows. Solid green lines show the timings of campaigns of IPT of school-aged children for a school-based delivery if campaigns occur once per school term and dashed green lines show the same but for monthly campaigns. Note that for the Central archetype, the optimal timing of the school term campaigns included two campaigns in the autumn term and none in the late spring term. The exact campaign times and school calendars are described in the appendix (pp 3-4). In the Sahel panel (far right), the orange dotted lines show timing of seasonal malaria chemoprevention campaigns; this is also the timing of community-based IPT of school-aged children rounds. Academic calendars from Zambia, the Democratic Republic of the Congo, and Burkina Faso were used to represent the Southern, Central, and Sahel archetypes, respectively.

Three transmission archetypes—Sahelian, Central African, and Southern African—were constructed to investigate how the effect of IPT in school-aged children might vary across the continent (figure 1). Each archetype was modelled with a unique annual vector habitat seasonality and vector species mix (appendix p 2). Within a given archetype, different background levels of transmission were explored (ranging from 5% to 40% *P falciparum* prevalence in children aged 2–10 years [*Pf*PR_2–10_]) by varying the total amplitude of available vector habitat.

### Intervention scenarios

Long-lasting insecticide-treated nets (ITNs) and clinical case management were always included as baseline interventions. In the Sahelian archetype, seasonal malaria chemoprevention for children younger than 5 years was also included. Appendix (pp 5–6) shows a detailed parametrisation of each baseline intervention. The baseline comparator scenario included no IPT of school-aged children. We also included a comparator scenario in which ITN coverage was increased from 70% to 90% but IPT of school-aged children was not introduced. Table 2, Table 3 show the full list of model scenarios.

**Table 2:** Model scenarios for Southern and Central transmission archetypes

	**Non-IPTsc interventions**	**IPTsc protocol**
1	Baseline health-seeking and ITN coverage	None
2	Baseline health-seeking; 90% ITN coverage	None
3	Baseline health-seeking and ITN coverage	School-based IPTsc with ASAQ each term
4	Baseline health-seeking and ITN coverage	School-based IPTsc with ASAQ each month
5	Baseline health-seeking and ITN coverage	School-based IPTsc with DP each term
6	Baseline health-seeking and ITN coverage	School-based IPTsc with DP each month
7–10	Baseline health-seeking and ITN coverage	Same as scenarios 3–6 but with test-and-treat instead of presumptive treatment
11–14	Baseline health-seeking and ITN coverage	Same as scenarios 3–6 but with ivermectin administered alongside the IPTsc drug
15–18	Baseline health-seeking and ITN coverage	Same as scenarios 3–6 but with primaquine administered alongside the IPTsc drug

ASAQ=artesunate–amodiaquine. DP=dihydroartemisinin–piperaquine. INT=insecticide-treated net. IPTsc=intermittent preventive treatment of school-aged children.

**Table 3:** Model scenarios for Sahel transmission archetype

	**Non-IPTsc interventions**	**IPTsc protocol**
1	Baseline health-seeking, SMC with SPAQ for children younger than 5 years, and ITN coverage	None
2	Baseline health-seeking, SMC with SPAQ for children younger than 5 years; 90% ITN coverage	None
3	Baseline health-seeking, SMC with SPAQ for children younger than 5 years, and ITN coverage	School-based IPTsc with SPAQ each term
4	Baseline health-seeking, SMC with SPAQ for children younger than 5 years, and ITN coverage	School-based IPTsc with SPAQ each month
5	Baseline health-seeking, SMC with SPAQ for children younger than 5 years, and ITN coverage	School-based IPTsc with DP each term
6	Baseline health-seeking, SMC with SPAQ for children younger than 5 years, and ITN coverage	School-based IPTsc with DP each month
7	Baseline health-seeking, SMC with SPAQ for children younger than 5 years, and ITN coverage	SMC with SPAQ for children aged 5–10 years
8	Baseline health-seeking, SMC with SPAQ for children younger than 5 years, and ITN coverage	SMC with SPAQ for children aged 5–15 yeas
9	Baseline health-seeking, SMC with SPAQ for children younger than 5 years, and ITN coverage	SMC with DP for children aged 5–10 years
10	Baseline health-seeking, SMC with SPAQ for children younger than 5 years, and ITN coverage	SMC with DP for children aged 5–15 years
11–14	Baseline health-seeking, SMC with SPAQ for children younger than 5 years, and ITN coverage	Same as scenarios 3–6 but with test-and-treat instead of presumptive treatment
15–18	Baseline health-seeking, SMC with SPAQ for children younger than 5 years, and ITN coverage	Same as scenarios 3–6 but with ivermectin administered alongside the IPTsc drug
18–21	Baseline health-seeking, SMC with SPAQ for children younger than 5 years, and ITN coverage	Same as scenarios 3–6 but with primaquine administered alongside the IPTsc drug

DP=dihydroartemisinin–piperaquine. INT=insecticide-treated net. IPTsc=intermittent preventive treatment of school-aged children. SMC=seasonal malaria chemoprevention. SPAQ=sulfadoxine–pyrimethamine–amodiaquine.

We typically modelled IPT of school-aged children as presumptive treatment of all children who are covered by the intervention. However, for comparison we also included scenarios in which IPT of school-aged children was performed as test-and-treat, wherein rapid diagnostic tests are used to identify active infections and only children who are positive by this test are treated.

### IPT of school-aged children drugs

We included scenarios for three of the most widely studied drug options: dihydroartemisinin–piperaquine, artesunate–amodiaquine, and sulfadoxine–pyrimethamine–amodiaquine. Artemether–lumefantrine was not considered in this analysis due to its widespread use as first-line therapy and its short duration of efficacy. We also explored the additional benefit of combining the primary IPT of school-aged children drug with primaquine or ivermectin, which target onward transmission through different mechanisms. The detailed pharmacokinetic and pharmacodynamic parametrisations of each drug as well as dose, route, and frequency of administration are discussed in the appendix (pp 5–6). Dihydroartemisinin–piperaquine and sulfadoxine–pyrimethamine–amodiaquine were the options considered for the Sahelian archetype. In the Central and Southern archetypes, dihydroartemisinin–piperaquine and artesunate–amodiaquine were considered due to high observed levels of sulfadoxine–pyrimethamine resistance in these regions (Table 2, Table 3)**.**

### Delivery mode: school-based versus community-based

School-based delivery of IPT in school-aged children was an option in all archetypes. In this delivery mode, campaigns occur either monthly or once per school term (three campaigns per year). In the Sahelian archetype, in which seasonal malaria chemoprevention is an established community-based intervention, there was also the option for community-based delivery of IPT of school-age children by extending seasonal malaria chemoprevention campaigns to either age 10 or 15 years (table 3). In areas where seasonal malaria chemoprevention is established, both community and school-based delivery of chemoprevention to older children might be referred to as extended-age seasonal malaria chemoprevention. However, for simplicity, we used the terminology of school-based delivery of IPT in school-aged children.

School-based IPT of school-aged children is administered only when school is in session. Optimal campaign timing differs by archetype depending on transmission seasonality, academic calendar, and campaign frequency (figure 1). The school calendars of Burkina Faso, the Democratic Republic of the Congo, and Zambia were used to represent their respective archetypes (see appendix [p 4] for additional details about the school calendars). Minor academic calendar variations among countries were not anticipated to substantially affect the results of the present study.

Treatment adherence was assumed to be higher for school-based IPT of school-aged children since all treatment doses can be directly observed. Adherence for community-based IPT of school-aged children is 80%, as for standard seasonal malaria chemoprevention. Adherence for school-based IPT of school-aged children is assumed to be 95%.

We assumed 60% coverage for each round of community-based IPT of school-aged children, as for standard seasonal malaria chemoprevention. Conversely, the coverage of school-based IPT depends on assumed school attendance rates. We assumed that 80% of children aged 6–12 years attend primary school,^29^ with smaller percentages of older children also attending (see appendix [p 8] for the assumed age distribution of children attending primary school and appendix [p 8] for the effect of IPT of school-aged children under differing school attendance levels). Of the children who do attend primary school, we assumed 90% coverage in each school-based IPT campaign round.

### Role of the funding source

The funder of the study had no role in study design, data collection, data analysis, data interpretation, or writing of the report.

## Results

**Figure 2:**
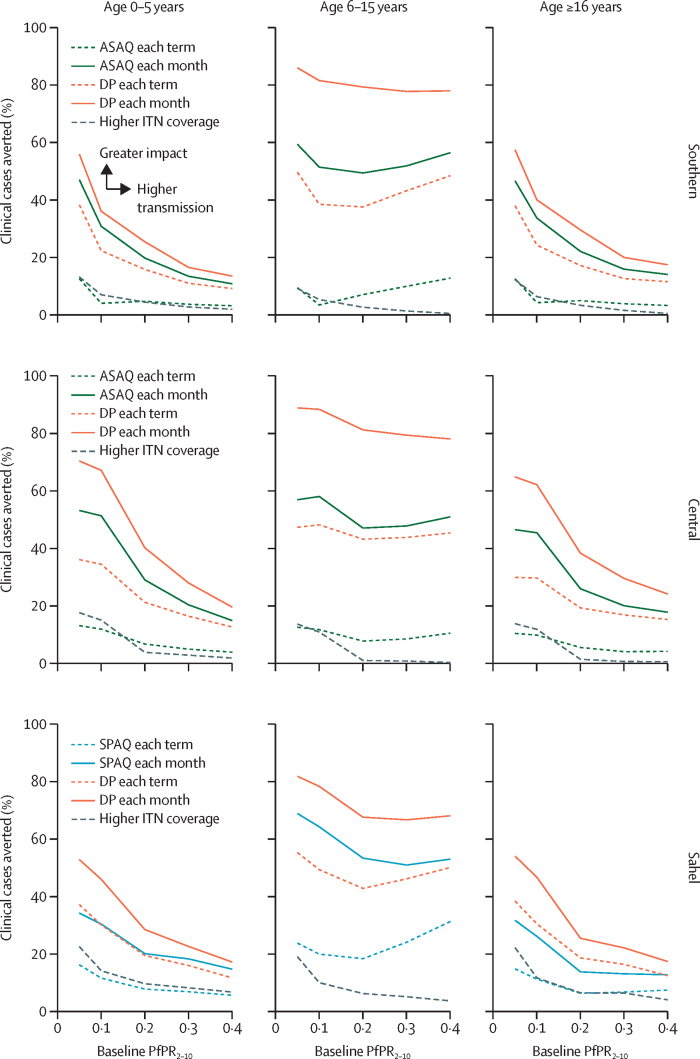
Effect of variations of IPT of school-aged children across transmission archetypes and age groups, compared with the scenario of no-IPT of school-aged children Percentage of cases averted is shown for the second year of the intervention. Blue and green curves show 14-day drug, orange curves show 28-day drug; lines shows campaign frequency of once per term (dashed) or once per month (solid). All simulations have the same baseline interventions of ITNs and health-seeking; the Sahel archetype also includes seasonal malaria chemoprevention. The dotted grey line shows, for comparison, the effect of increasing the ITN coverage from 70% to 90%. The effect of IPT in school-aged children is a function of both drug duration and campaign frequency. IPT of school-aged children can substantially reduce clinical burden in school-age children. Community benefits are most pronounced at low levels of transmission (*Plasmodium falciparum* parasite rate in children aged 2–10 years [*Pf*PR_2–10_] 5–10%). IPT=intermittent preventive treatment**.** ASAQ=artesunate–amodiaquine. DP=dihydroartemisinin–piperaquine. ITNs=insecticide-treated nets. SPAQ=sulfadoxine–pyrimethamine–amodiaquine.

2 years after introduction of IPT in school-aged children, clinical cases were reduced in this group, younger children, and adults across all archetypes and transmission levels (figure 2; see appendix [p 8] for baseline annual clinical incidence in the absence of IPT in school-aged children). IPT in school-aged children induced strong direct benefit, with up to approximately 80% of cases averted in this age group (up to about 2 cases per child per year), depending on the intensity of the IPT protocol. In general, each IPT protocol averted a similar proportion of cases in school-aged children independent of transmission level. By contrast, IPT of school-aged children averted a higher proportion of clinical cases in other age groups as the level of transmission decreased; 20–60% of clinical cases were averted in younger children and adults (up to about 0·6 cases per person per year), with a larger proportion of cases averted at lower levels of transmission (*Pf*PR_2–10_ 5–10%). Overall, the effect of IPT in school-aged children on transmission decreased as the transmission level increased (figure 2). For comparison, increasing ITN coverage from 70% to 90% generally averted less than 10% of cases (a mean of about 70 cases per 1000 in the population).

**Figure 3:**
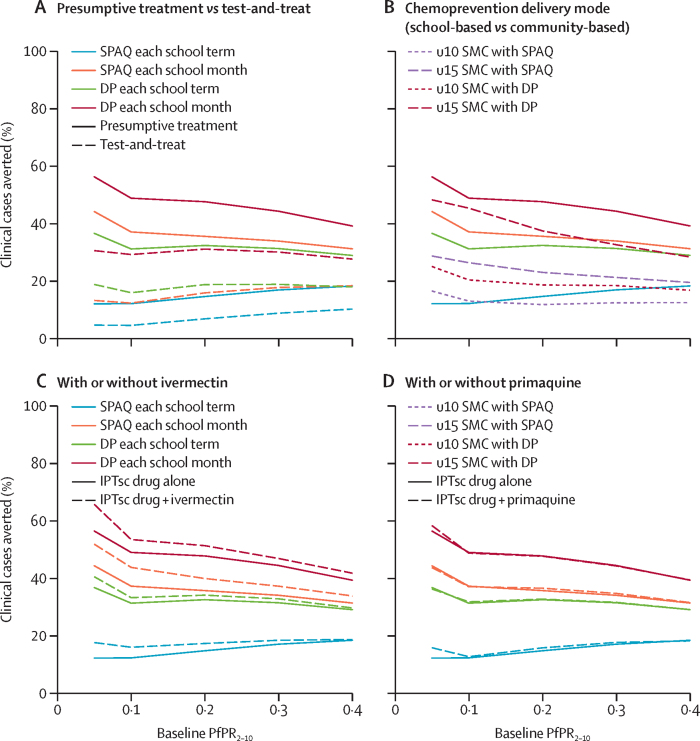
Effects of protocol variations in IPT of school-aged children in the Sahel archetype Percentage of cases averted is shown for all ages, in the second year of the intervention. (A) A presumptive treatment approach versus an approach of treating only children who are positive by rapid detection test. (B) School-based chemoprevention for school-age children (solid lines) versus extending seasonal chemoprevention to school-age children (dashed and dotted lines). (C and D) The effect of IPT of school-aged children with or without an additional transmission-targeted drug included. Broadly, results are similar for the other two transmission archetypes; see appendix p 9 for comparison. IPT=intermittent preventive treatment DP=dihydroartemisinin–piperaquine. PfPR_2–10_=*Plasmodium falciparum* parasite rate in children aged 2–10 years. SMC=seasonal malaria chemoprevention. SPAQ=sulfadoxine–pyrimethamine–amodiaquine. u10=children aged 10 years and younger. u15=children aged 15 years and younger.

The effect of IPT in school-aged children depended on both duration of drug efficacy and campaign frequency (figure 2). The most intensive protocol, monthly campaigns with dihydroartemisinin–piperaquine, the longest-acting drug combination evaluated, averted 40–75% of clinical cases in the population (ranging from 350 to 900 annual cases per 1000, depending on background transmission level). This protocol averted 70–90% of clinical cases in school-aged children (up to 2·0 cases per child per year) and 20–60% of cases in other age groups (up to about 0·6 cases per person per year) depending on the background level of transmission. When delivered monthly, the shorter-acting drugs (sulfadoxine–pyrimethamine–amodiaquine in the Sahelian archetype or artesunate–amodiaquine in the Central and Southern archetypes) averted 30–50% of cases in the population (ranging from 300 to 600 annual cases per 1000, depending on background transmission level); this protocol averted 40–60% of cases in school-aged children (up to about 1·3 cases per child per year) and 15–50% of cases in other age groups (up to about 0·5 cases per person per year). Shorter-acting drugs delivered monthly had a greater effect than did dihydroartemisinin–piperaquine delivered once per school term. Across all transmission archetypes, a test-and-treat approach was substantially less effective than presumptive treatment; a monthly test-and-treat approach of IPT in school-aged children averted about the same number of cases as presumptive treatment each term (figure 3, appendix p 10).

The addition of single low dose primaquine in each IPT of school-aged children campaign round did not substantially affect the number of clinical cases (figure 3). When administered monthly in the Central and Southern archetypes, the addition of ivermectin as an endectocide increased the indirect effect of IPT in school-aged children, especially at low levels of transmission (appendix p 9). For example, when ivermectin was added to monthly artesunate–amodiaquine in these archetypes at 5% *Pf*PR_2–10_, the proportion of cases averted compared with the scenario of no-IPT in school-aged children scenario increased from 50% (about 630 cases averted per 1000) to approximately 80% (about 1000 cases per 1000).

In the Sahelian archetype, the effect of IPT in school-aged children also depended on the mode of delivery (figure 3). School-based delivery of sulfadoxine–pyrimethamine–amodiaquine each school term averted 20–30% of cases in school-aged children (mean 0·4 cases per child per year), which was similar to the effect of extending community-based delivery of seasonal malaria chemoprevention with sulfadoxine–pyrimethamine–amodiaquine up to age 10 years. School-based delivery of sulfadoxine–pyrimethamine–amodiaquine each month averted more cases (50–70% of school-aged children cases averted, mean 0·9 cases per child per year) than extending seasonal malaria chemoprevention community-based delivery of sulfadoxine–pyrimethamine–amodiaquine up to age 15 years (35–40% of school-aged children cases averted, mean 0·6 cases per child per year). School-based delivery of dihydroartemisinin–piperaquine each term averted 40–55% of cases among school-aged children (mean 0·8 cases per child per year), compared with 25–30% of cases in school-aged children averted by extending seasonal malaria chemoprevention with dihydroartemisinin–piperaquine up to age 10 years (mean 0·5 cases per child per year) and 45–65% averted by extending to age 15 years (mean 0·9 cases per child per year). Overall, school-based delivery of monthly dihydroartemisinin–piperaquine had the greatest effect of all modelled IPT of school-aged children protocols, averting 70–80% of clinical cases in school-age children (mean 1·2 cases per child per year).

## Discussion

This analysis performed with a detailed mathematical model of malaria transmission suggests that across diverse transmission settings, IPT of school-aged children could substantially reduce clinical malaria not only in school-aged children but also in other age groups due to reduction of transmission. The model results suggest that the greatest benefits of adding IPT of school-aged children to current malaria control interventions occur when duration of protection is maximised, either with a long-lasting drug or through frequent campaigns. Under these conditions, IPT of school-aged children averted approximately half or more of clinical cases in this group and averted 20–40% of cases in other age groups. The direct benefit of this intervention to school-aged children themselves is roughly constant across transmission levels, while the indirect, community benefit of IPT of school-aged children is larger at lower levels of transmission.

We compared IPT of school-aged children to enhanced vector control under the assumption of baseline 70% ITN coverage; this baseline reflects the WHO recommendation that all at-risk populations maintain effective vector control.^30^ IPT of school-aged children with dihydroartemisinin–piperaquine or sulfadoxine–pyrimethamine–amodiaquine each month or dihydroartemisinin–piperaquine each term averted more cases than enhancing ITN coverage from 70% to 90%, in both school-aged children and in other age groups. The lower effect of ITNs in the model is due, in part, to the discarding of bednets over time.^31^

IPT of school-aged children can be delivered through schools or through community-based campaigns. Schools provide a convenient delivery point given increasing school enrolment rates across sub-Saharan Africa.^32^ However, in strongly seasonal settings where standard seasonal malaria chemoprevention is already being implemented, an alternative delivery mode of IPT of school-aged children is to extend seasonal malaria chemoprevention to older ages, an option already under consideration in some countries.^33^ Differences between the two approaches include campaign timing and adherence. Seasonal malaria chemoprevention is designed to be carried out during the peak transmission season, whereas school-based programmes can be carried out only during the school year; this latter difference is important if most of the transmission season occurs while school is out of session. Adherence rates might differ between delivery modes; for example, a recent school-based pilot of IPT in school-aged children achieved very high rates of directly observed therapy since doses were given by schoolteachers in their classrooms.^34^

Mathematical transmission models are useful, low-cost tools to predict intervention effect and guide investment decisions. Nevertheless, all models have important limitations and are not a replacement for rigorous clinical trials. The detailed realism of the agent-based model used in this study incurs costs in terms of model simplicity and computational complexity. Unfortunately, an exhaustive sensitivity analysis is computationally infeasible; instead, we have kept fixed the core model parameters, which best fit a wide range of gold-standard datasets and focused on model scenarios tailored to study the effect of IPT of school-aged children. Reported uncertainties in intervention effects are, therefore, stochastic uncertainties under fixed best-fit model parameters; this approach might underestimate the true model uncertainty when marginalised over all model parameters.

This study focuses on estimating epidemiological effect and does not include cost or cost-effectiveness analyses, which will be key to guide programmatic decisions. The outputs presented in this paper, particularly age-specific and setting-specific estimates of cases averted, provide a strong foundation for future modelling studies that integrate costing data, delivery strategies, and health-system constraints.

Despite these limitations, our findings align with and expand upon the two previous modelling studies related to IPT of school-aged children. Stuckey and colleagues^22^ predict a small transmission decrease under the addition of screen-and-treatment with artemether–lumefantrine for school-aged children twice per school term in a low transmission setting in western Kenya; this prediction accords with our finding that drug efficacy duration is a key factor in the effect of IPT of school-aged children. Runge and colleagues^23^ modelled IPT of school-aged children administered twice per year with a hypothetical drug with a 14-day duration in moderate-to-high transmission areas of Tanzania. The researchers found that when added to existing vector control, IPT of school-aged children resulted in a 15% reduction in clinical cases in the total population. Our model predicted a similar effect for artesunate–amodiaquine and sulfadoxine–pyrimethamine–amodiaquine administered once per school term, suggesting consistency across models despite fundamentally different model architectures.

There are potential risks of IPT of school-aged children that we have not directly addressed. Although the drugs we investigated have established safety profiles, large-scale use might reveal rare side-effects. Another concern with widespread chemoprevention is increased selective pressure, which might accelerate development of drug resistance. To date, only one study has reported a correspondence between IPT of school-aged children and elevated drug resistance markers,^35^ although a recent reanalysis of these data found no evidence of selection for resistance.^36^ Strategies to mitigate selection for resistant parasites include mosaic or mixing approaches with the use of drug regimens with different or antagonistic mechanisms of drug resistance, targeting specific age strata to restrict the extent of drug use, or the addition of drugs that target individual-level transmission in case of breakthrough infection (eg, primaquine or ivermectin). In the future, vaccines or monoclonal antibodies might prove to be superior options to target school-aged children; if these products show similar direct benefit, then similar levels of community benefit might also be expected.

There are also potential benefits to IPT of school-aged children that we have not analysed. Given its large direct benefit to school-aged children, IPT might improve cognitive outcomes and educational achievements,^18^ which might lead to long-term societal benefits and increased human capital.^37^ School-based delivery platforms could be more cost-effective and sustainable than mass campaigns, especially if delivery is shared with parallel school-based interventions (eg, deworming, school feeding, and human papillomavirus vaccination).^38^, ^39^ As control programmes expand vaccine and chemoprevention coverage in young children, clinical burden might shift to school-aged children. Developing interventions to target this age group will put malaria control efforts ahead of the shifting epidemiological curve.

We note and echo the WHO guidance that the addition of IPT of school-aged children should not compromise interventions to maximise prevention in younger children (younger than 5 years), who are at highest risk of severe disease.^30^ Although IPT of school-aged children does indirectly reduce clinical burden (and hence severe disease) in younger children through community benefit, chemoprevention or vaccination of younger children is still expected to reduce severe disease more than IPT of school-aged children. IPT of school-aged children should be considered only once appropriate case management and prevention measures for younger children are established.

### Contributors

## Data sharing

All code is available on Github at https://github.com/jgsuresh/malaria-sac-ipt.

## Declaration of interests

We declare no competing interests.
