# Supplementary material for: Effect of malaria chemoprevention for school-age children across transmission archetypes: a modelling study
Source: Lancet Glob Health. 2025 Nov 12;13(12):e2144–52. doi: 10.1016/S2214-109X(25)00325-0 (PMC12621301; doi:10.1016/S2214-109X(25)00325-0)
Supplement: Supplementary appendix [file mmc1.pdf]

THE LANCET
Global Health

Supplementary appendix

This appendix formed part of the original submission and has been peer reviewed. We post it as supplied by the authors.

Supplement to: Suresh J, Zimmermann M, Maiteki C, et al. Effect of malaria chemoprevention for school-age children across transmission archetypes: a modelling study. *Lancet Glob Health* 2025; **13:** e2144–52.

Supplementary Information for:

Modeling the impact of malaria chemoprevention for school-age children across transmission archetypes

Joshua Suresh, PhD[1], Marita Zimmermann, PhD[1], Catherine Maiteki, MSc[2], Anne Stahlfeld, MPH[3], Abigail Pratt, MSc[4], Prof Don P. Mathanga, PhD[5], Prof Sarah G. Staedke, PhD[6], Prof Miriam K. Laufer, MD[7], Prof Chris Drakeley, PhD[8], Caitlin Bever, PhD[1]*, Lauren M. Cohee, MD[6,7]*

[1]Institute for Disease Modeling, Bill and Melinda Gates Foundation, Seattle, WA USA

[2]Ministry of Health, Kampala, Uganda

[3]The Feinberg School of Medicine, Northwestern University, Chicago, IL USA

[4]Bill and Melinda Gates Foundation, Seattle, WA USA

[5]Malaria Alert Centre, Kamuzu University of Health Sciences, Blantyre, Malawi

[6]Liverpool School of Tropical Medicine, Liverpool UK

[7]Center for Vaccine Development and Global Health, University of Maryland School of Medicine, Baltimore, MD USA

[8]London School of Hygiene and Tropical Medicine, London UK

* shared last author

Supplemental Text 1: Transmission archetypes

We modeled the impact of IPTsc in three transmission archetypes, each of which is meant to roughly represent the seasonality and transmission properties of a broad region of sub-Saharan Africa. For each archetype, the total transmission level can be adjusted by increasing or decreasing the mean amount of larval habitat available throughout the year.

Table S1. Transmission archetype details

**Archetype**	**Vector species (and approximate abundance)**	**Vector characteristics**	**Assumed academic calendar**
Sahel	Gambiae (100%)	Indoor feeding fraction = 90%<br>Anthropophily = 65%	Burkina Faso: Major school vacation from beginning of June to mid-September [1]
Central	Funestus (5%)<br>Gambiae (95%)	Indoor feeding fraction for both species = 50%<br>Funestus anthropophily = 65%<br>Gambiae anthropophily = 85%	Democratic Republic of the Congo: Major school vacation between July and September [2]
Southern	Arabiensis (85%)<br>Funestus (15%)	Arabiensis indoor feeding fraction = 50%<br>Funestus indoor feeding fraction = 90%<br>Anthropophily for both species = 65%	Zambia: Major school vacations from early December to mid-January and mid-April to mid-May [3]
[1] https://publicholidays.africa/burkina-faso/school-holidays/<br>[2] https://www.eduquepsp.education/v1/wp-content/uploads/2022/07/CALENDRIER-SCOLAIRE-2022-2023_EPST.pdf<br>[3] https://www.educationinzambia.com/school-calendar-2017-2020/			

Table S2. Campaign timing for different transmission archetypes and campaign frequencies

		Transmission archetype		
		**Sahel**	**Central**	**Southern**
**Campaign timing**	**Once per term**	1. Jan 2nd<br>2. May 31st<br>3. Oct 5th	1. March 1st<br>2. Sept 21st<br>3. Dec 15th	1. Jan 15th<br>2. June 14th<br>3. Sept 11th
	**Once per month**	1. Jan 15th<br>2. Feb 15th<br>3. March 15th<br>4. April 15th<br>5. May 15th<br>6. Sept 15th<br>7. Oct 15th<br>8. Nov 15th<br>9. Dec 15th	1. Jan 5th<br>2. Feb 4th<br>3. March 1st<br>4. April 1st<br>5. May 1st<br>6. June 1st<br>7. July 1st<br>8. Sept 1st<br>9. Oct 1st<br>10. Nov 1st<br>11. Dec 1st	1. Jan 15th<br>2. Feb 15th<br>3. Mar 15th<br>4. April 15th<br>5. May 15th<br>6. June 15th<br>7. July 15th<br>8. August 15th<br>9. Sept 1st<br>10. Oct 1st<br>11. Nov 1st<br>12. Dec 1st

Supplemental Text 2: School calendars

To better understand the overlap of transmission season and school calendar for each transmission archetype, we performed a search for publicly available academic calendars across the sub-Saharan continent. Academic calendars vary in their details from country to country, so there is no single academic calendar for each region. Instead, we selected specific country academic calendars that are a reasonable representation of countries in the same transmission region (see Figure 1). For the Southern archetype, we use Zambia's school calendar, which has longer holidays from December to mid-January and again from mid-April to mid-March. For the Central archetype, we use that of the Democratic Republic of Congo, which has a major school vacation between July and September. For the Sahel archetype, we use Burkina Faso's calendar, which has a long school holiday from early June to mid-September, covering most of the rainy season.

Supplemental Text 3: Intervention parametrization

Each simulation includes a 70% coverage distribution of long-lasting ITNs. In the Central and Southern archetype, the distribution of these nets occurs at the beginning of January in the first simulation year. In the Sahel archetype, the net distribution takes place in late June of the first simulation year, just before the start of the transmission season. Net usage follows a seasonal pattern and varies by age, with individuals between the ages of 5 and 20 only 65% as likely to use their ITN as younger children and older adults. The duration of ITN efficacy is also strongly influenced by net discarding rates [1]. We assume that 60% of individuals discard their nets quickly, with an exponential decay timescale of 260 days. The other 40% of individuals retain their nets much longer, with a discard timescale of 2100 days. These parameters are derived from rich net retention data obtained during a mass drug administration (MDA) trial conducted in Zambia [2] and they are consistent with estimated net retention rates observed in various countries across sub-Saharan Africa [1]. ITNs are assumed to have an initial killing efficacy of 60%; this efficacy wanes with an exponential decay timescale of 4 years. ITNs are also assumed to have an initial blocking efficacy of 90%; this efficacy wanes with an exponential decay timescale of 2 years.

Along with ITNs, case management of symptomatic infections is a baseline intervention in all simulated scenarios. The presence of clinical symptoms in an infection is determined by parasite load and host immunity, following a detailed within-host process [3]. If symptoms arise, the infected individual has some probability of seeking care from the health system, depending on the individual's age and the severity of the symptoms. This probability is defined as the case management rate. Case management rates are assumed to be 60% for uncomplicated malaria cases in individuals under 5 years of age and 30% in individuals over 5 years of age. Case management rates for severe cases are assumed to be higher: 90% in individuals under 5 years of age and 80% in individuals over 5 years of age. We explore the impact of IPTsc under different health-seeking rates in Figure S6.

In the Sahel archetype, SMC for children under 5 is an additional baseline intervention. SMC is implemented in four annual rounds, with 60% coverage in each round. The SMC drugs are sulfadoxine + pyrimethamine + amodiaquine (SPAQ). A course of SPAQ involves three daily doses (see below for more details); we assume 80% adherence to the drug regimen: 80% of everyone who receives the first drug dose goes on to take the second dose, and 80% of those go on to take the final dose.

Drug pharmacokinetic and pharmacodynamic (PK/PD) parameters for dihydroartemisinin piperaquine (DP) and primaquine are drawn from the calibrations performed by Gerardin et al [4]. We assume the same PD parameters for artesunate (AS) as for dihydroartemisinin. PD parameters for AQ were determined from clinical trial data of both AQ and ASAQ in Tanzania [5] and ASAQ from Tororo, Uganda [6]. PK parameters for SP were drawn from Bell et al [7]; PD parameters were then inferred by fitting to an observational study of SMC efficacy from Baba et al [8]. Ivermectin is assumed to last for 3 days in the bloodstream, during which 95% of blood-feeding vectors die immediately afterwards; the assumed drug efficacy and duration yields a similar effect size to that observed in a recent ivermectin mass drug administration trial [9].

The full adult treatment dose for DP is 120mg dihydroartemisinin + 960mg piperaquine, taken orally over a three-day regimen. DP dosing is age-dependent; children under 7.3 years old receive 3/4th of the adult dose; 7.3-9.4 year olds receive 0.875 of the adult dose; children above this age receive the full adult dose. The adult dose for SP is 1500mg sulfadoxine + 75mg pyrimethamine, taken orally in one sitting. SP dosing is age-dependent; children under 1 year old receive 1/8th of the adult dose; children ages 1-5 years old receive 1/4th of the adult dose. The adult dose for amodiaquine is 600mg, taken orally over a three-day regimen. AQ dosing is age-dependent; children under 1 year old receive 0.376 of the adult dose; children ages 1-5 receive 0.44 of the adult dose. The adult dose for artesunate (taken with amodiaquine in the combination drug ASAQ) is 450mg artesunate, taken orally over a

three-day regimen. AS dosing is age-dependent; children under 2 years of age receive 0.167 of the adult dose; children 2-5 years old receive 0.33 of the adult dose. The full adult treatment dose for primaquine is 210 mg, taken orally over a 14-day regimen. Primaquine dosing is age-dependent; children under 5 years old receive one sixth of the adult dose; 5-9 year olds receive a third of the adult dose; 9-14 year olds receive two thirds of the adult dose; children above this age receive the full adult dose. In the model, ivermectin is not modeled with a PK/PD approach, but is rather assumed to have the same vector-killing efficacy regardless of age.

Supplemental Text 4: Transmission-targeting drugs

In some settings, ivermectin may be a helpful drug to include with IPTsc. Figures 3 and S3 shows the additional impact of including the transmission-targeted drugs ivermectin or primaquine to IPTsc. We find almost no benefit to adding primaquine; this accords with previous modeling work which found that primaquine is only beneficial in mass drug administration campaigns when performed with very high coverage and in very low transmission settings [4]. Conversely, adding ivermectin to IPTsc does increase overall impact, especially at lower transmission and with more frequent IPTsc campaigns [9,10]. Figures 3 and S3 shows that at fixed transmission levels, ivermectin is more effective when the seasonality of transmission is less pronounced. This seemingly paradoxical result arises due to the convention we have used of reporting averaging transmission over the year. For example, in a comparison between simulations from the Sahel and Central archetypes that have the same annualized prevalence, the Sahel simulations have a much higher level of vector biting during the transmission season than ever occurs in the Central simulations; this high level of population-level biting, combined with the fact that ivermectin is only being given to school-age children and not the entire population, reduces the additional impact of including ivermectin.

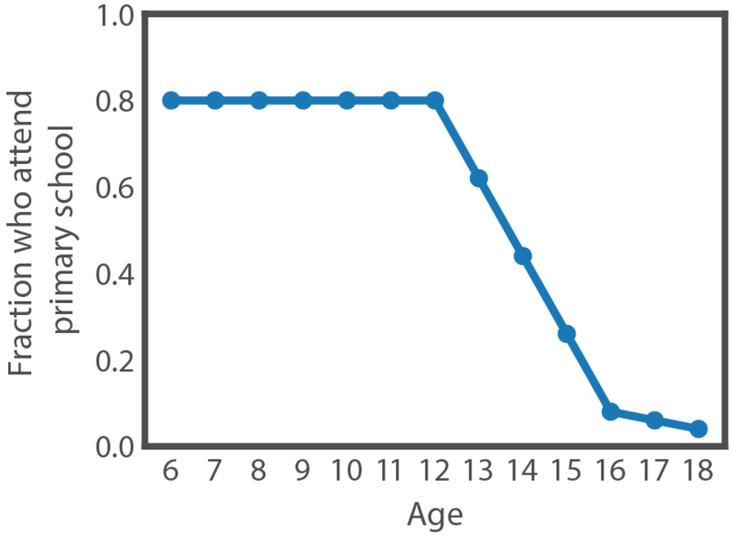

Figure S1 Primary school attendance by age. We assume that 80% of all children ages 6-12 attend school, with decreasing primary school attendance from ages 13-18.

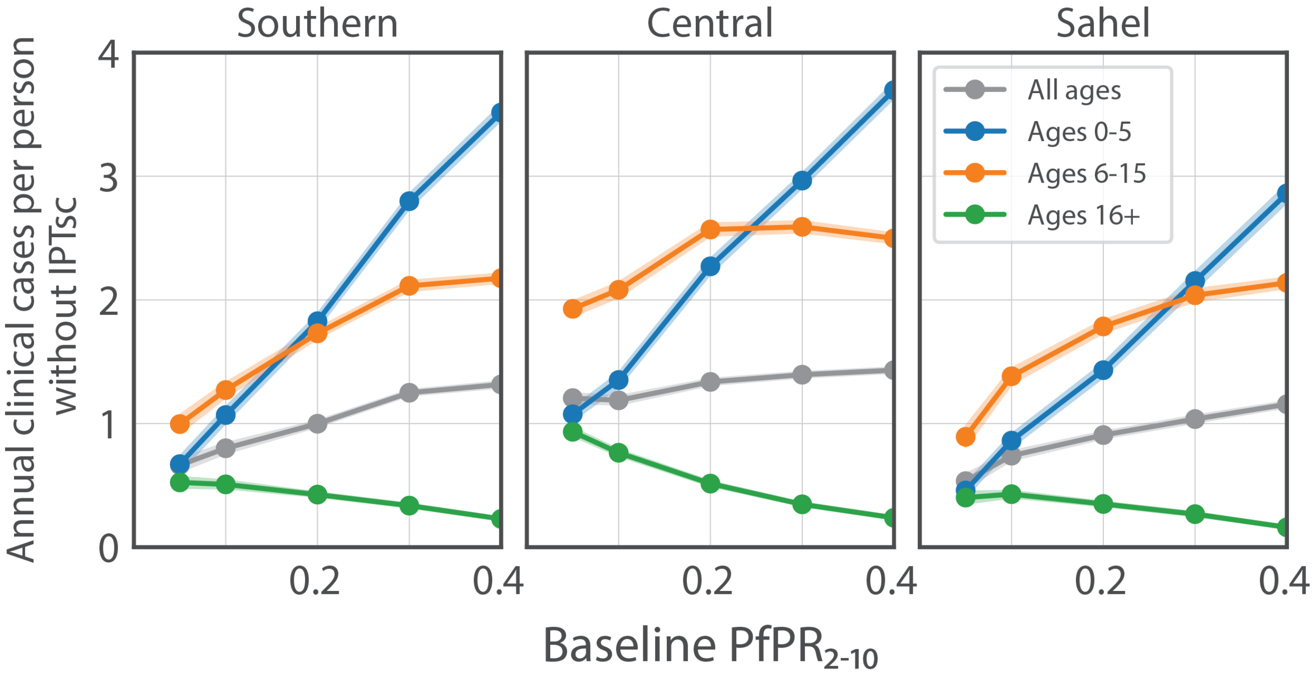

Figure S2 Annual clinical incidence without IPTsc. Clinical incidence in modeled population with baseline interventions but no IPTsc. Note that this is the rate of clinical cases in the population, not the number of cases that would be reported based on the assumed health-seeking rate. Baseline interventions are bednets and health-seeking in all archetypes; SMC for children under 5 in Sahel archetype. Solid lines show the mean annual clinical incidence for each age group, over 100 simulation replicates. Shaded regions 1 standard deviation spread around the mean.

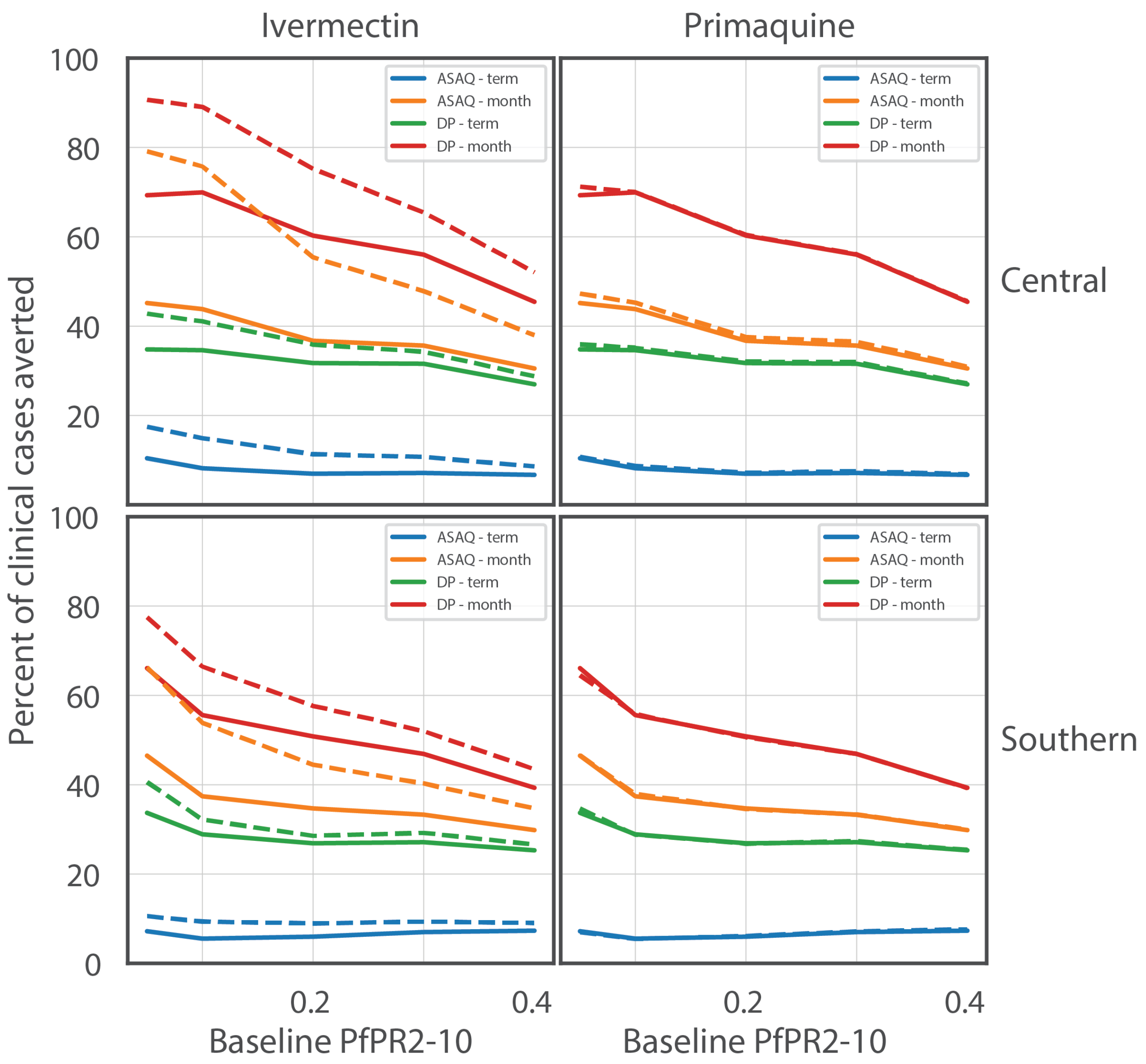

Figure S3 Impact of adding transmission-targeting drugs to IPTsc regimen in Central and Southern archetypes. Horizontal axis shows the baseline prevalence before including IPTsc; vertical axis shows percent of clinical cases averted in the overall population. Solid lines show the IPTsc protocol without the additional drug, dashed lines show with the additional drug. See Figure 3 for corresponding results for Sahel archetype. Ivermectin (left column) increases IPTsc impact under low levels of transmission, frequent IPTsc campaigns, and less pronounced seasonality. Primaquine does not add much benefit to any of the modeled IPTsc protocols.

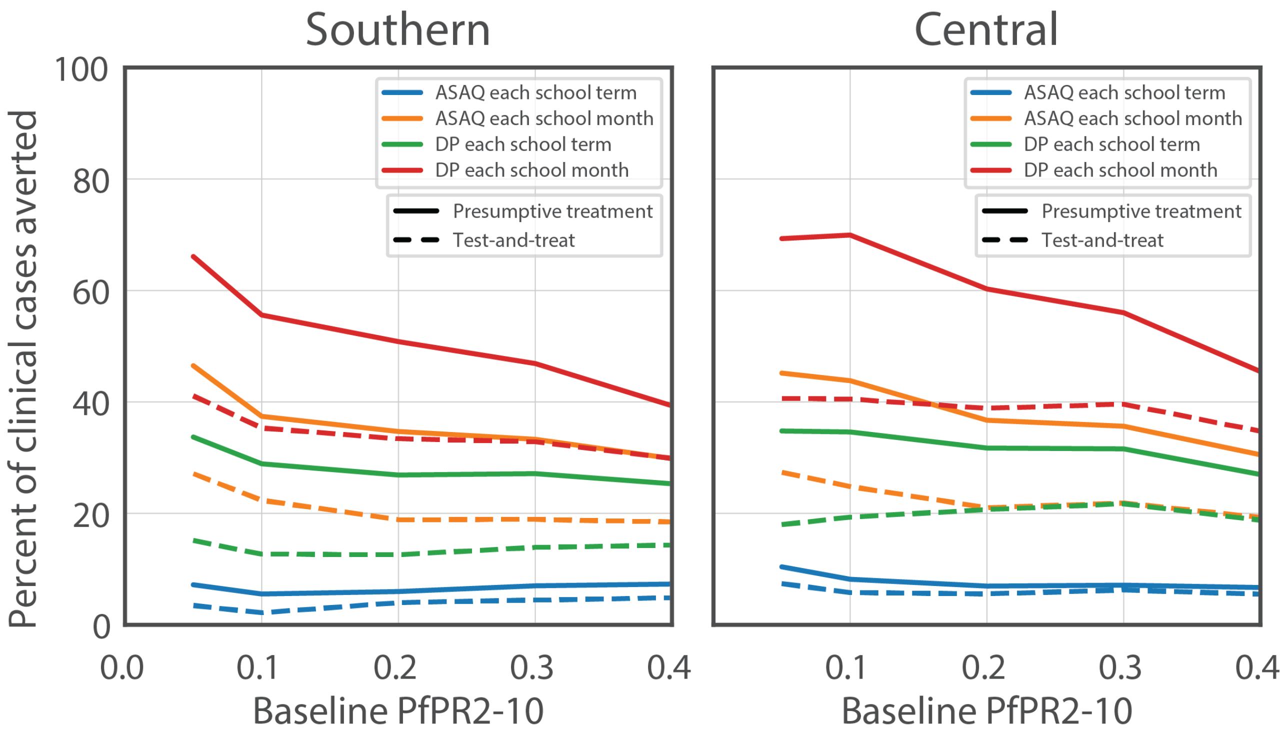

Figure S4 Impact of test-and-treat IPTsc versus presumptive treatment in Central and Southern archetypes. Horizontal axis shows the baseline prevalence before including IPTsc; vertical axis shows percent of clinical cases averted in the overall population. Solid lines show the IPTsc protocol with presumptive treatment, dashed lines show with test-and-treat. See Figure 3 for corresponding results for Sahel archetype. Monthly test-and-treat averts roughly the same number of cases as presumptive treatment each school term.

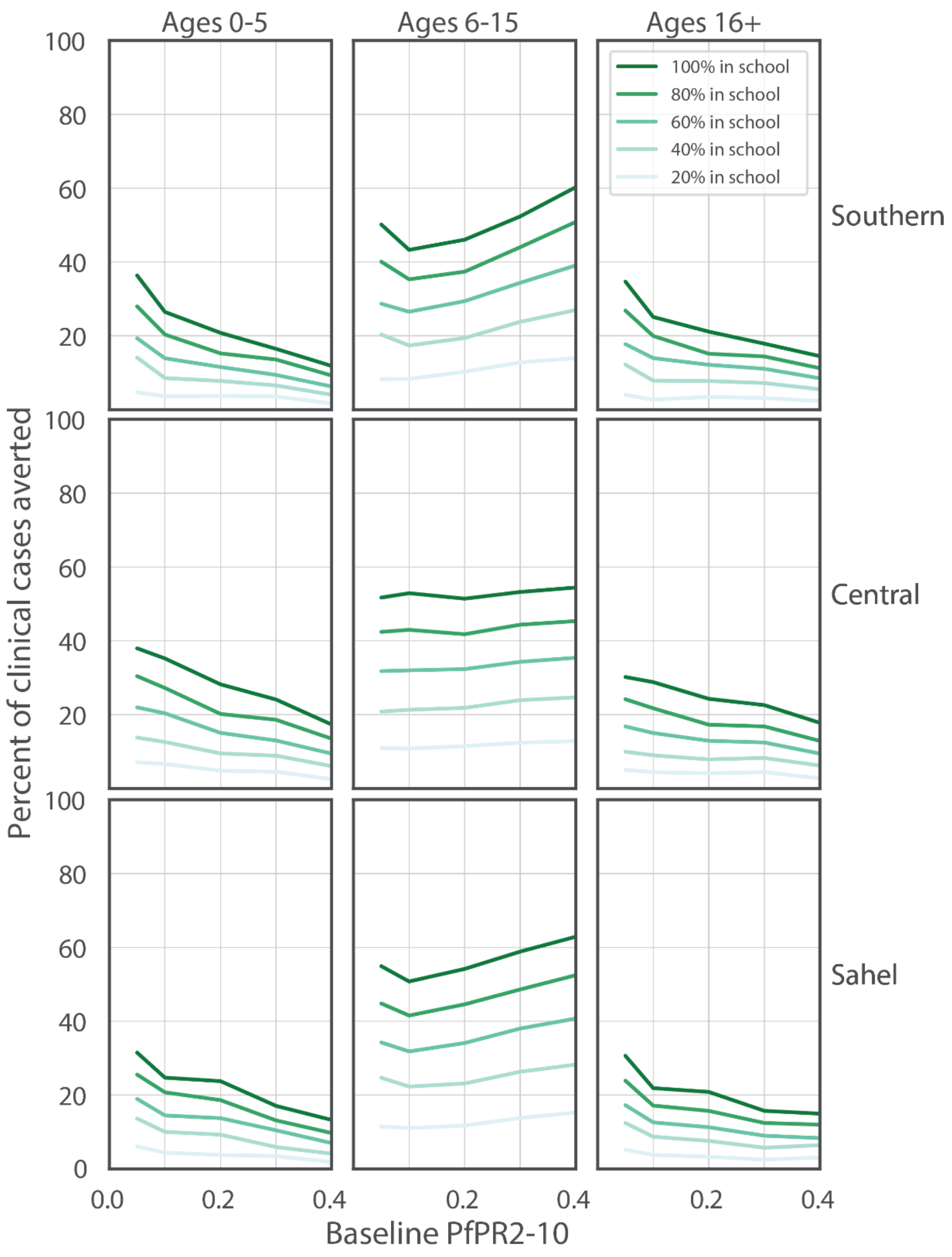

Figure S5 IPTsc impact under different school attendance rates. IPTsc protocol is assumed to be DP once per school term.

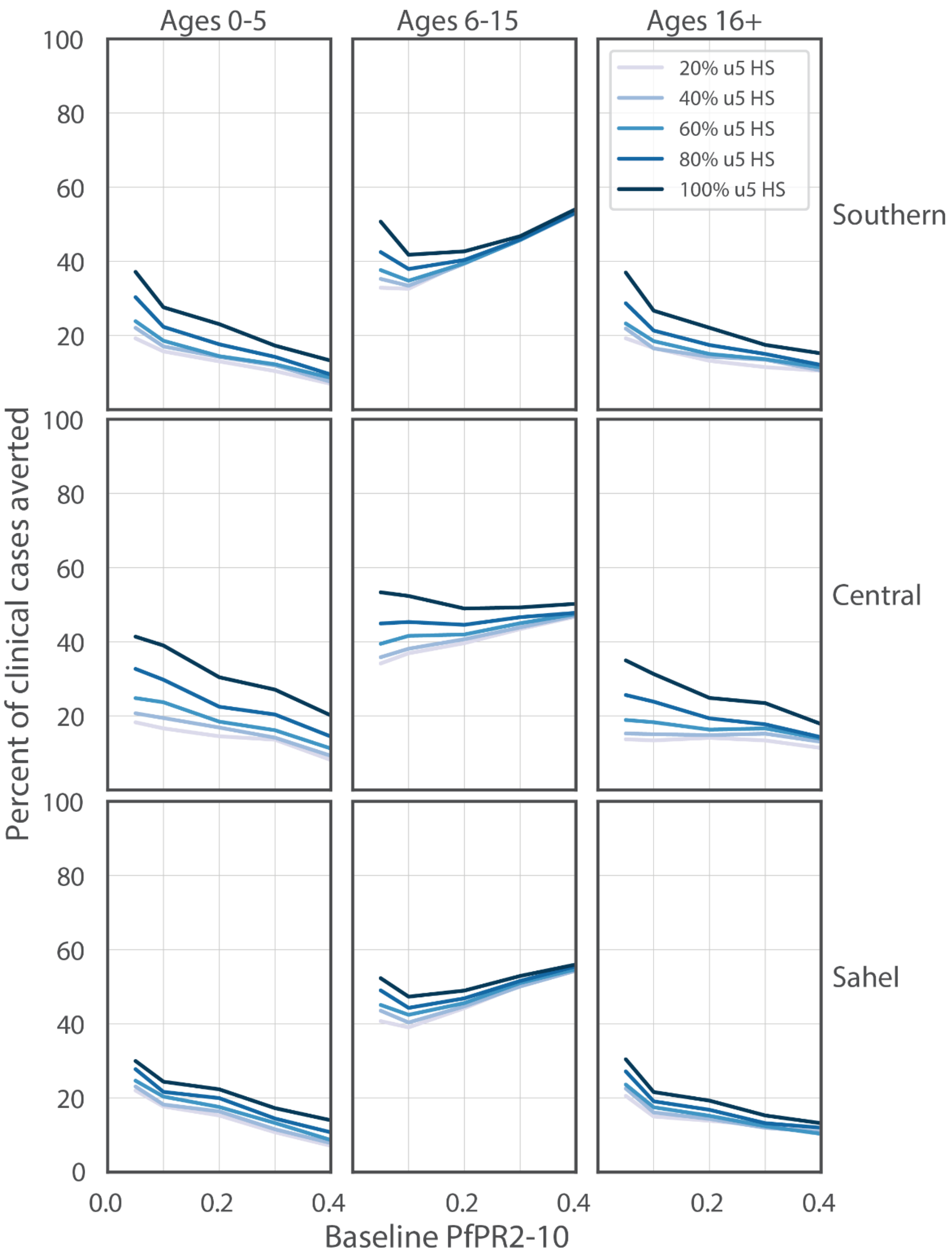

Figure S6 IPTsc impact under different health-seeking rates. IPTsc protocol is assumed to be DP once per school term.

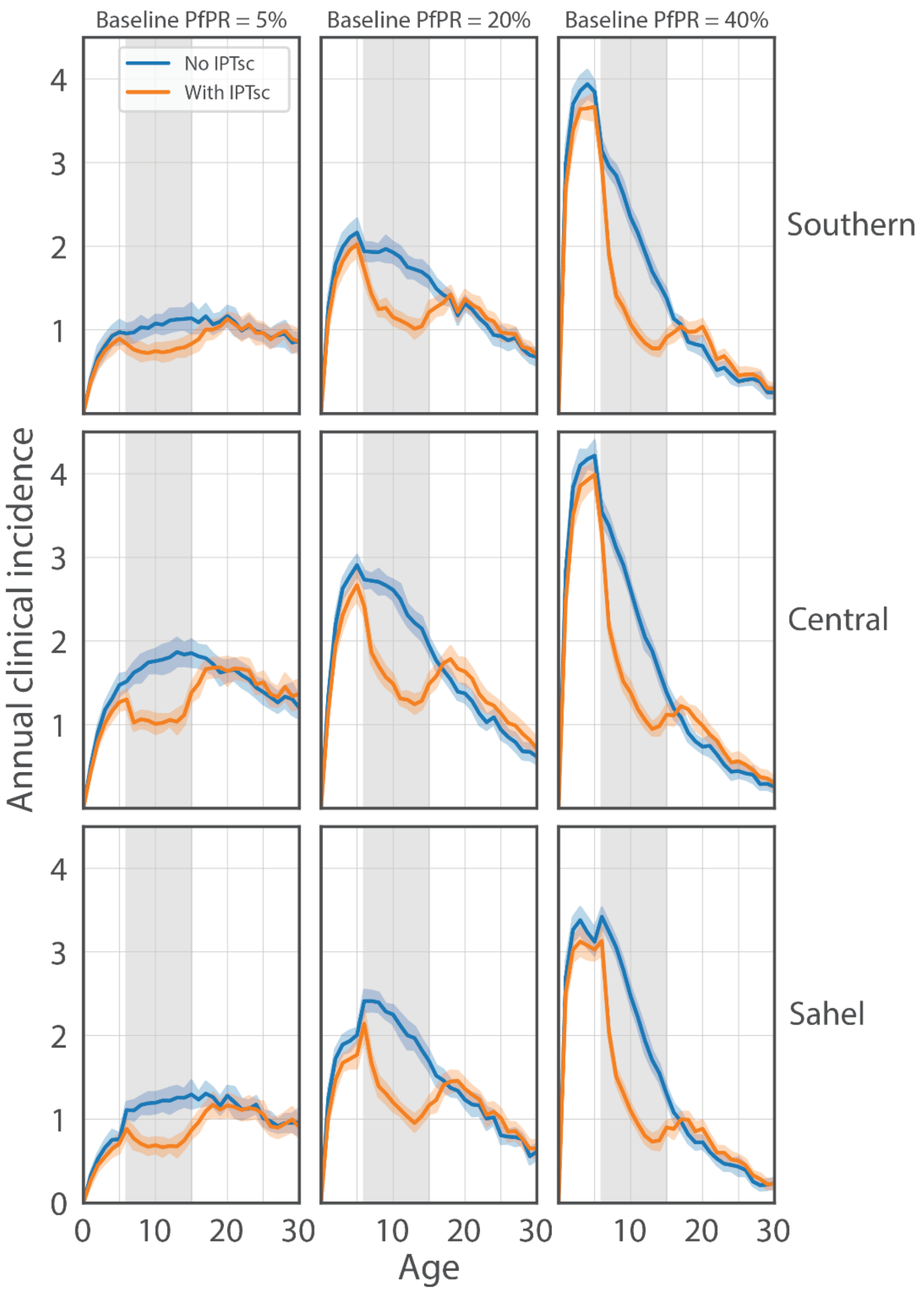

Figure S7 Clinical incidence in individuals who age out of long-term IPTsc. Annual clinical incidence by age, comparing long-term IPTsc against no-IPTsc. Panels show different transmission archetypes (rows) and intensities (columns). The blue curve shows a population who receives baseline interventions but no IPTsc; the orange curve shows a population which has received 20 years of annual IPTsc. IPTsc protocol is DP every school term. Shaded colored regions shows standard deviation spread across 20 simulation replicates. Shaded gray region shows assumed age range for IPTsc. For individuals aging out of long-term IPTsc, the model predicts little case "rebound". At low transmission intensity, there is no rebound. At higher transmission intensity, individuals aging out of IPTsc have slightly elevated clinical incidence (about 10% or less) for a few years than they would have if they had never received IPTsc.

References

1. Bertozzi-Villa A, Bever CA, Koenker H, et al. Maps and metrics of insecticide-treated net access, use, and nets-per-capita in Africa from 2000-2020. Nature Communications 2021 12:1 [Internet]. Nature Publishing Group; **2021** [cited 2023 Jan 16]; 12(1):1–12. Available from: https://www.nature.com/articles/s41467-021-23707-7

2. Eisele TP, Bennett A, Silumbe K, et al. Short-term Impact of Mass Drug Administration With Dihydroartemisinin Plus Piperaquine on Malaria in Southern Province Zambia: A Cluster-Randomized Controlled Trial. J Infect Dis [Internet]. Oxford Academic; **2016** [cited 2023 Feb 2]; 214(12):1831–1839. Available from: https://academic.oup.com/jid/article/214/12/1831/2632617

3. Eckhoff P. P. falciparum Infection Durations and Infectiousness Are Shaped by Antigenic Variation and Innate and Adaptive Host Immunity in a Mathematical Model. PLoS One [Internet]. **2012**; 7(9):e44950. Available from: https://www.ncbi.nlm.nih.gov/pmc/articles/PMC3446976/

4. Gerardin J, Eckhoff P, Wenger EA. Mass campaigns with antimalarial drugs: a modelling comparison of artemether-lumefantrine and DHA-piperaquine with and without primaquine as tools for malaria control and elimination. BMC Infect Dis [Internet]. **2015**; 15:144. Available from: https://www.ncbi.nlm.nih.gov/pmc/articles/PMC4376519/

5. Mutabingwa TK, Anthony D, Heller A, et al. Amodiaquine alone, amodiaquine+sulfadoxine-pyrimethamine, amodiaquine+artesunate, and artemether-lumefantrine for outpatient treatment of malaria in Tanzanian children: a four-arm randomised effectiveness trial. Lancet [Internet]. Lancet; **2005** [cited 2023 Jan 16]; 365(9469):1474–1480. Available from: https://pubmed.ncbi.nlm.nih.gov/15850631/

6. Yeka A, Banek K, Bakyaita N, et al. Artemisinin versus Nonartemisinin Combination Therapy for Uncomplicated Malaria: Randomized Clinical Trials from Four Sites in Uganda. PLoS Med [Internet]. Public Library of Science; **2005** [cited 2023 Jan 16]; 2(7):e190. Available from: https://journals.plos.org/plosmedicine/article?id=10.1371/journal.pmed.0020190

7. Bell D, Nyirongo S, Mukaka M, Molyneux M, Winstanley P, Ward S. Population Pharmacokinetics of Sulfadoxine and Pyrimethamine in Malawian Children With Malaria. Clin Pharmacol Ther [Internet]. **2011**; 89(2):268–275. Available from: https://onlinelibrary.wiley.com/doi/abs/10.1038/clpt.2010.297

8. Baba E, Hamade P, Kivumbi H, et al. Effectiveness of seasonal malaria chemoprevention at scale in west and central Africa: an observational study. The Lancet [Internet]. Lancet Publishing Group; **2020** [cited 2023 Jan 16]; 396(10265):1829–1840. Available from: http://www.thelancet.com/article/S0140673620322273/fulltext

9. Selvaraj P, Suresh J, Wenger EA, Bever CA, Gerardin J. Reducing malaria burden and accelerating elimination with long-lasting systemic insecticides: a modelling study of three potential use cases. Malar J [Internet]. BioMed Central; **2019** [cited 2023 Jan 16]; 18(1):307. Available from: /pmc/articles/PMC6727392/

10. Slater HC, Walker PGT, Bousema T, Okell LC, Ghani AC. The potential impact of adding ivermectin to a mass treatment intervention to reduce malaria transmission: A modelling study. Journal of Infectious Diseases. Oxford University Press; **2014**; 210(12):1972–1980.
